# In memory of James Taylor: the birth of Galaxy

**DOI:** 10.1186/s13059-020-02016-0

**Published:** 2020-04-30

**Authors:** Anton Nekrutenko, Michael C. Schatz

**Affiliations:** 1 Department of Biochemistry and Molecular Biology, The Pennsylvania State University, University Park, PA USA; 2 Departments of Computer Science and Biology, Johns Hopkins University, Baltimore, MD USA

James Peter Taylor, the Ralph S. O’Connor Professor of Biology and Computer Science at Johns Hopkins University (JHU), passed away on April 2, 2020. He was 40 years old. James was an exceptional scientist, colleague, mentor, and community builder, who worked at the intersection of biology and computer science. His life’s pursuit was to understand how genomic and epigenomic information is processed during normal development and dysregulated in disease. As co-leader of the Galaxy (http://galaxyproject.org) [1] and AnVIL (http://anvilproject.org) Projects, a major thrust of James’ career aimed to support the work of others, especially to empower those with limited resources. His impact was broad, as innumerable scientists worldwide benefited from his leadership, mentoring, and scientific contributions.

James completed a BS. in Computer Science (Magna Cum Laude) at the University of Vermont in 2000. After spending 3 years as a senior software engineer in Vermont, he joined the Ph.D. program in Computer Science at the Pennsylvania State University in the Center for Comparative Genomics and Bioinformatics under the supervision of Professors Webb Miller and Francesca Chiaromonte. His Ph.D. work focused on developing novel machine learning approaches for identifying functional elements in the genome [2], leveraging the wealth of new data from ENCODE (https://www.encodeproject.org/) and the alignments of newly assembled vertebrate genomes [3].

## Creating the Galaxy

During this time at Penn State, James worked closely with me (Anton Nekrutenko) and others to start the Galaxy Project, a comprehensive web platform for open and reproducible computational data analysis. The first software commit to the project repository was on June 1, 2005, by James. Today, Galaxy needs no introduction to anyone working in genomics, but at the time was a major advance above the ad hoc command line bioinformatics analysis that dominated the field. The initial version introduced support for accessing remote data resources and visualizing the results [4]. The project grew to incorporate thousands of analysis tools into one unified graphical user interface, accessible to anyone via a web browser. The Galaxy Project remains a landmark achievement and has forever changed the way scientists analyze and share data.

After completing his Ph.D. in 2006, James worked for 2 years as a visiting member of the Courant Institute for Mathematical Sciences at New York University. During these 2 years, Galaxy became one of his principal projects as James coded several of its iconic features, including the three-pane interface, the “noodly” workflow editor, and the dynamic genome browser Trackster. In 2008, James started his laboratory at Emory University in the Department of Biology and the Department of Mathematics & Computer Science. He was promoted to Associate Professor with tenure in 2013, shortly before his move to JHU where he was promoted to Professor in 2018.

It was at Emory University, and later JHU, where the Galaxy platform exploded in popularity, driven by the growth of high-throughput sequencing data and large-scale cloud computing. This combination of technologies has proven to be transformative to the field, and the Galaxy Project has reached a wide audience of scientists evidenced by thousands of citations. Today, thousands of scientists around the world use Galaxy daily.

The Galaxy Project is not only a software platform but also a scientific community. James’ dedication to accessible, reproducible, and transparent research promoted a community of researchers that extended far beyond the original development team. Scientists, often working completely independently from the founders, have taken Galaxy into entire new research domains. The strength of the Galaxy community is also seen every year at the community-run conference that brings hundreds of participants together to share their latest contributions and applications.

James was an ardent and principled advocate for open science, especially open access to scientific data and open-source software. James said that software may come and go, including even Galaxy, but the metadata that Galaxy collects will ultimately be his most valuable contribution to science. This metadata enables anyone to observe all analysis steps and reproduce entire analyses, providing the bedrock for future discoveries. Without such transparency and rigor, he explained, the entire field will suffer.

## Beyond the Galaxy

While James is widely known for his development of the Galaxy Project, he made numerous other contributions to science and education. James collaborated with many experimentalists to analyze Hi-C data and interrogate the 3D organization of the genome. At Emory, James worked to understand how DNA-binding proteins shape nuclear architecture as cell fates are specified [5]. At JHU, James collaborated to decipher the mechanisms of the mysterious “A” and “B” compartmentalization of the genome in relation to the nuclear lamina [6]. He applied his knowledge of the genome to identify “buttons” that promote gene regulation between chromosomes [7]. James’ curiosity and collegiality led to new scientific directions as he evaluated ribosome profiling data to identify A and P sites [8], analyzed long-read transcriptome data to characterize development in worms [9], and interpreted transcriptome data from human retinal organoids to understand how our eyes develop [10], among many other projects. No matter the question, approach, or organism posed to him, James was always up for a new scientific challenge.

James’ love of science was matched by his passion for teaching. At JHU, he completely transformed and revitalized the curriculum within the graduate biology program to require training in computational and quantitative research for all Ph.D. students. This was rooted in his recognition that all subfields of biology are increasingly data-rich and quantitative. For many of the students, James’ “boot camp” was their first introduction to such techniques; as with a military boot camp, the students were left mentally and physically exhausted at the end of the day. Ultimately, however, they all appreciated how it made them stronger and more empowered scientists. James’ passion for teaching extended to the annual Computational Genomics course at Cold Spring Harbor Laboratory that he co-taught with David Hawkins and William Pearson.

Galaxy, and every aspect of James’ life, benefited tremendously from James’ leadership style. Rather than an authoritarian top-down approach, he led by example, contributing code and technical oversight with exceptional mastery and precision. While he generally had a laid back personality, he was always willing to fight for his students, friends, and colleagues. Whenever I (Schatz) felt stuck about a particular administrative or logistical issue, James fired back about how the university administration should support us even more. I also knew that whenever I was not in the room, he would be there to advocate for me on my behalf. He offered these gifts to everyone, which promoted deep friendships and community for all of us around him.

## Thank you James

James was always kind, friendly, and generous, and we will miss him dearly. He is survived by his wife Meredith Greif, a sociology professor also at Johns Hopkins. We all will strive to carry on his vision in his absence.

If we could talk with him one last time, we would say: Thank you, James, on behalf of your community, for being a friend, a leader, a colleague, a mentor, a student, a scientist, a programmer, and an advocate. Thank you for embracing your bioinformatics community and sharing resources in the spirit of accessibility and scientific progress. Thank you for sharing your strengths in selfless ways and for dedicating your life’s work to further scientific research in genomics and beyond. Thank you for caring, participating, and leading the way. You leave behind many teams and communities, many people who worked on the things that you started, and many who thought that they would have more time to work with you on projects and reach new scientific heights. Please know that we will go forward and continue what you started, keeping you close, building on your work, and expanding on the many foundations that you provided. We will try to be better friends, leaders, colleagues, mentors, students, scientists, and advocates for each other, because we know, through your work and your many examples, that this is what you wanted us to do.
